# Economic resilience dataset in facing physical distancing during COVID-19 global pandemic

**DOI:** 10.1016/j.dib.2020.106069

**Published:** 2020-07-25

**Authors:** Muhammad Fitri Rahmadana, Gaffar Hafiz Sagala

**Affiliations:** Faculty of Economics, Universitas Negeri Medan, Indonesia

**Keywords:** COVID-19, Global pandemic, Economic resilience, Vulnerability, Household

## Abstract

The dataset was collected using the survey method with an electronic questionnaire. The use of electronic questionnaires is intended to reach many respondents during the physical distancing caused by the COVID-19 global pandemic. The instrument used in data collection was adapted from Alinovi, Mane, and Romano (2008). The instrument was designed anonymously to ensure the privacy and independence of respondents in giving their responses. Data that is captured includes several categories either nominal, ordinal, or interval refers to the information that needs to be captured. Determination of the type of data also refers to the recommendation of previous research. The data was collected using the Google form platform. Questionnaire distribution was conducted on April 12–20, 2020, and 1096 responses were collected. The date is two weeks after the government call of the Republic of Indonesia to carry out Physical Distancing to anticipate the COVID-19 Global Pandemic. The entire data is then screened and filtered so that it only leaves the data with respondents who are domiciled in Medan City. After filtered, there is remained 846 data that is ready for analysis. In order to make more informative data, researchers conducted a descriptive statistical analysis, ANOVA, Kruskal Wallis, and the Spearman's rank correlation. Analysis of the data provides valuable information related to the interrelation of each item and the pattern of economic resilience that the urban city community has as a data of the COVID-19 global pandemic. Researchers can then further analyzed the data with more advanced analytical tools to produce more valuable information in the development of science and in developing strategic policies related to anticipating the economic vulnerability of the household due to the global pandemic disaster.

Specifications table

**Table:** 

Subject	Social Science, Geography, Planning and Development; Economics, Econometrics and Finance
Specific subject area	Household Economic Resilience in Facing COVID-19 Global Pandemic
Type of data	Table
How data were acquired	The data were collected using a questionnaire that contains the items which measure the Resilience Dimensions of Household adapted from Alinovi, Mane, and Romano (2008) (Mendeley Data Appendix 1). That is the primary data were presented in the article (Mendeley Data Appendix 2). Researchers collecting the data using electronic questionnaire which distributed from all of the social media platforms to acquire data.
Data format	Raw, Filtered, and Analyzed
Parameters for data Collection	The data was collected from the population facing the government instructions to implement Physical Distancing in order to anticipate the spread of COVID-19. Data were collected two weeks after the instructions first announced. This is intended to get an actual response after the respondent feels the impact of the application of physical distancing. The unit of analysis is the household.
Description of data collection	The instrument was designed to as much as possible to capture the actual conditions faced by respondents in the form of perception. The type of data collected is nominal, ordinal, interval, and ratio depending on the indicator characteristic in question. The questionnaire was distributed electronically using Google Form to reach a broad response. The electronic questionnaire was distributed massively to all social media networks. Respondents were asked to fill out the questionnaire voluntarily. Collecting data is done in one week.
Data source location	Medan, North Sumatra, Indonesia
Data accessibility	The data is accessible in Mendeley Data, DOI: http://dx/doi.org/10.17632/2jtn5dcnvd.1

## Value of the data

• This data is useful for observing patterns of household economic resilience in the face of a global pandemic, which in this case is COVID-19. In the future this pattern will be useful in anticipating pandemics that may occur with certain social engineering so that household economic stability can be maintained.
• This data will be useful for policymakers in determining economic risk control strategies that can attack households when implementing physical distancing in the face of any disaster, especially the COVID-19 global pandemic. On the other hand, other researchers can also use this data to develop knowledge and recommendation for more valuable strategies of mitigation.
• This data can be further analysed using more sophisticated statistical tools to investigate important variables that determine the economic resilience during Physical Distancing or the differences in responses from various sample groups, such as logistic regression, multinomial regression, and structural equational modelling (SEM). The data was possible for wide range data analysis because it has a vary data characteristics, like nominal, ordinal, and interval, also has a suitable sample size.
• The data presented respondents' perceptions regarding their wellbeing. It is indeed respondents' subjective wellbeing, but it represents the definition of their economic resilience, which is in line with the positivism paradigm. Therefore, it will more suitable for developing public-oriented regulation.
• Further analysis of this data will be useful in generating social engineering that has the opportunity to control the economic vulnerability of the household.

## 1 Data description

Data analysis that has been done is descriptive statistics, ANOVA, Kruskal Wallis, and Spearman's Rank correlation [1,2]. Descriptive statistics indicate that each sample category represents the observed conditions, such as physical distancing patterns, sources of family income, and estimates of the duration of economic resilience. Thus, the data have useful variations. The demography of respondents on gender shows that female is dominant respondents. Meanwhile, in the marital category, the status is dominated by respondents who have the status of singles, married, and married with children. And in the educational background of respondents was dominated by the Senior High School Education and Diploma/Bachelor. These conditions seem to bias to gender and marital status, but actually, the level of analysis was household instead of the individual. Then, the respondent not represents himself but his family. We controlled the objectivity of data using two items in the questionnaire, that is 1) Number of family members and 2) Family income estimation. Related to a family member, we have cross-tabulate the number of family income with marital status. From 841 data, there are only 27 respondents who are single and have no family members other than himself (See Table 1). A lot of respondents who have a single marital status probably happen because he or she is a member of the family as children instead of parents. It is possible, and we control the maturity of a respondent using the age that is 18th years old as a minimum.

**Table 1:** Cross-Tabulation between Marital Status and Number of Family Member.

Marital Status	Number of Family Member					Total
	0	1–2	3–4	5–6	>6	
Married with Children	0	18	89	45	12	164
Married	0	74	125	79	20	298
Single	27	71	102	147	39	386
Divorce	0	4	1	0	0	5
Divorce with Children	0	5	6	3	0	14
Total	27	171	323	274	71	867

Based on the data presented in Table 2, the respondents mostly did the physical distancing in level strict and moderate. While very few respondents did not carry out physical distancing. Then, on the aspect of the source of the family's main income, the group of respondents who have the monthly salary as a civil servant, military, police, pensioner, or private employee represent half of the respondents, while the rest are scattered in other groups, namely business or entrepreneurship, workers with daily/uncertain income, workers with weekly income, and workers with project-based income. This distribution is actually already good because in general, it is divided into two categories, that is employee workers, and non-employees. Furthermore, in the estimation of economic resilience during the physical distancing period, the sample was evenly distributed from each of the offered schemes, namely <2 weeks, 2–4 weeks, 4–12 weeks, and> 12 weeks.

**Table 2:** Descriptive Statistics of Characteristics Respondent.

	Gender		Age	Marital Status					Educational Background					
	Male	Female		Married with child/ children	Married	Single	Widow/ Widower	Widow/ Widower with Child/ Children	Primary School	Junior High School	Senior High School	Diploma/ Bachelor	Master Degree	Doctoral
Pattern of Physical Distancing														
Implement Social distancing strictly (*N* = 248)	*N* = 81% = 32.7	*N* = 167% = 67.3	Mean = 31,45SD = 11.59	*N* = 49%= 19.8	*N* = 87% = 35.1	*N* = 109% = 44.0	*N* = 0% = 0.0	*N* = 3% = 1.2	*N* = 0% = 0.0	*N* = 3% = 1.2	*N* = 98% = 39.5	*N* = 92% = 20.6	*N* = 51% = 20.6	*N* = 4% = 1.6
Social/Physical Distancing with Outdoor/Outside Activity (*N* = 594)	*N* = 260% = 43.8	*N* = 334% = 56.2	Mean = 32.40SD = 11.61	*N* = 111% = 18.7	*N* = 203% = 34.2	*N* = 266% = 44.8	*N* = 3% = 0.5	*N* = 11% = 1.9	*N* = 12% = 2.0	*N* = 15% = 2.5	*N* = 146% = 41.4	*N* = 226% = 38.0	*N* = 87% = 14.6	*N* = 8% = 1.3
Do not apply Social distancing (*N* = 25)	*N* = 11% = 44.0	*N* = 14% = 56.0	Mean = 29.24SD = 10.03	*N* = 4% = 16.0	*N* = 8% = 32.0	*N* = 11% = 44.0	*N* = 2% = 8.0	*N* = 0% = 0.0	*N* = 1% = 4.0	*N* = 3% = 12.0	*N* = 12% = 48.0	*N* = 7% = 28.0	*N* = 2% = 8.0	*N* = 0% = 0.0

Source of the family's main income														
Monthly salary as a civil servant, military, police, pensioner, or private employee (*N* = 467)	*N* = 187% = 40.0	*N* = 280% = 60.0	Mean = 32.68SD = 11.07	*N* = 101% = 21.6	*N* = 161% = 34.5	*N* = 199% = 42.6	*N* = 1% = 0.2	*N* = 5% = 1.1	*N* = 2% = 0.4	*N* = 4% = 0.9	*N* = 125% = 26.8	*N* = 203% = 43.5	*N* = 123% = 26.3	*N* = 10% = 2.1
Business or Entrepreneur-ship (*N* = 144)	*N* = 56% = 38.9	*N* = 88% = 61.1	Mean = 28.20SD = 9.83	*N* = 22% = 15.3	*N* = 45% = 31.3	*N* = 73% = 50.7	*N* = 2% = 1.4	*N* = 2% = 1.4	*N* = 1% = 0.7	*N* = 1% = 0.7	*N* = 82% = 56.9	*N* = 51% = 35.4	*N* = 8% = 5.6	*N* = 1% = 0.7
Workers with daily/uncer-tain income (informal workers) (*N* = 170	*N* = 71% = 41.8	*N* = 99% = 58.2	Mean = 34.25SD = 13.55	*N* = 30% = 17.6	*N* = 61% = 35.9	*N* = 74% = 43.5	*N* = 1% = 0.6	*N* = 4% = 2.4	*N* = 7% = 4.1	*N* = 11% = 6.5	*N* = 105% = 61.8	*N* = 45% = 26.5	*N* = 2% = 1.2	*N* = 0% = 0.0
Workers with weekly income (*N* = 41)	*N* = 17% = 41.5	*N* = 24% = 58.5	Mean = 31.00SD = 12.37	*N* = 7% = 17.1	*N* = 15% = 36.6	*N* = 16% = 39.0	*N* = 1% = 2.4	*N* = 2% = 4.9	*N* = 3% = 7.3	*N* = 4% = 9.8	*N* = 28% = 68.3	*N* = 5% = 12.2	*N* = 0% = 0.0	*N* = 1% = 2.4
Workers with project-based income (*N* = 45)	*N* = 21% = 46.7	*N* = 24% = 53.3	Mean = 30.29SD = 10.05	*N* = 4% = 8.9	*N* = 16% = 35.6	*N* = 24% = 53.3	*N* = 0% = 0.0	*N* = 1% = 2.2	*N* = 0% = 0.0	*N* = 1% = 2.2	*N* = 16% = 35.6	*N* = 21% = 46.7	*N* = 7% = 15.6	*N* = 0% = 0.0

If Physical Distancing will continue to apply, how long will you and your family survive economically														
< 2 weeks (*N* = 190)	*N* = 62	*N* = 128	Mean = 31.90	*N* = 32	*N* = 71	*N* = 82	*N* = 3	*N* = 2	*N* = 4	*N* = 10	*N* = 96	*N* = 68	*N* = 11	*N* = 1
	% = 32.6	% = 67.4	SD = 11.59	% = 16.8	% = 37.4	% = 43.2	% = 1.6	% = 1.1	% = 2.1	% = 5.3	% = 50.5	% = 35.8	% = 5.8	% = 0.5
2 – 4 Weeks (*N* = 282)	*N* = 113	*N* = 169	Mean = 31.89	*N* = 43	*N* = 99	*N* = 135	*N* = 1	*N* = 4	*N* = 7	*N* = 9	*N* = 131	*N* = 98	*N* = 36	*N* = 1
	% = 40.1	% = 59.5	SD = 11.89	% = 15.2	% = 35.1	% = 47.9	% = 0.4	% = 1.9	% = 2.5	% = 3.2	% = 46.5	% = 34.8	% = 12.8	% = 0.4
4 – 12 Weeks (*N* = 204)	*N* = 94	*N* = 110	Mean = 31.48	*N* = 40	*N* = 71	*N* = 88	*N* = 0	*N* = 5	*N* = 1	*N* = 1	*N* = 67	*N* = 93	*N* = 38	*N* = 4
	% = 46.1	% = 53.9	SD = 10.87	% = 19.6	% = 34.8	% = 43.1	% = 0	% = 2.5	% = 0.5	% = 0.5	% = 32.8	% = 45.6	% = 18.6	% = 2.0
> 12 Weeks (*N* = 191)	*N* = 83	*N* = 108	Mean = 33.00	*N* = 49	*N* = 57	*N* = 81	*N* = 1	*N* = 3	*N* = 1	*N* = 1	*N* = 62	*N* = 66	*N* = 55	*N* = 6
	% = 43.5	% = 56.5	SD = 11.82	% = 25.7	% = 29.8	% = 42.4	% = 0.5	% = 1.6	% = 0.5	% = 0.5	% = 32.5	% = 34.6	% = 28.8	% = 3.1

Furthermore, Table 3 shows the difference in responses that the group of respondents had from the resilience variables analyzed. The significance of differences indicated by the number of *p*-values <0.05. Analysis of this data was carried out with ANOVA and Kruskal Wallis [1]. Based on the number of p-values of each item, the data indicate that the group of respondents who conducted physical distancing in a strict, moderate manner, and did not carry out physical distancing did not differ in aspects of income and access to food, access to basic services, social safety nets, and assets. However, it shows differences in adaptive capacity and stability in some items. Meanwhile, in contrary, groups of respondents who were differentiated based on the source of main income and the estimated duration of economic resilience showed a significant difference almost across items both in income and food access variables, access to basic services, social safety nets, assets, adaptive capacity, and stability. Some things that need to be highlighted are the increase in the water bill, access to assistance, and the number of families did not show significant differences either in respondents who are distinguished based on physical distancing patterns, sources of income, and the estimated duration of economic resilience. This pattern indicates that the water consumption pattern is relatively constant in any community conditions. While aid or loan is not a very crucial aspect for all sample categories, but other expertise that possible to make it economically benefit is an important aspect. Finally, the number of families does not become a differentiator, perhaps because of the large number of family members followed by a large number of economically productive families. The data shows the attractiveness of patterns and characteristics of the Medan community in terms of their economic resilience.

**Table 3:** Comparative Analysis Related to Pattern of Physical Distancing, Source of the Main Income and Economic Resilience.

	Pattern of Social/Physical Distance		Source of the family's main income		If Physical Distancing will continue to apply, how long will you and your family survive economically	
	F-test; Chi-Square	Sig	F-test; Chi-Square	Sig	F-test; Chi-Square	Sig
Income and Food Access						

Income	0.462	0.630	4.238	0.002**	14.249	0.000**
Number of Family Members	1.242	0.289	13.367	0.000**	0.794	0.497
Income Per-capita	2.561	0.078	223.092	0.000**	21.247	0.000**
Shopping pattern in accommodating the daily food needs under normal circumstances	2.581	0.275	47.581	0.000**	16.174	0.001**
Shopping patterns during Physical Distancing	20.213	0.000**	46.926	0.000**	42.387	0.000**

Access to Basic Services						

Type of health services	3.639	0.162	76.363	0.000**	41.270	0.000**
Quality of health services	8.674	0.013*	42.787	0.000**	17.523	0.001**
Quality of the Education System	4.568	0.102	47.639	0.000**	8.002	0.046*
Mobility disrupted during Physical Distancing	4.006	0.135	27.471	0.000**	10.253	0.017*
Transportation restrictions during Physical Distancing	18.661	0.000**	10.917	0.028*	5.309	0.151
Access to clean water during Physical Distancing	2.538	0.281	13.915	0.008**	9.274	0.026*
Ability to pay water bills during Physical Distancing	6.768	0.034	111.489	0.000**	111.291	0.000**
Increasing water bill during Physical Distancing	2.619	0.270	4.851	0.303	1.322	0.724
Getting water bill subsidy during Physical Distancing	0.660	0.719	18.920	0.001**	7.001	0.072
Access to electricity during Physical Distancing	5.118	0.077	7.391	0.117	34.339	0.000**
Ability to pay electricity bills during Physical Distancing	2.513	0.285	104.385	0.000**	123.387	0.000**
Increasing electricity bill during Physical Distancing	6.568	0.037*	16.113	0.003**	7.221	0.065
Getting electricity bill subsidy during Physical Distancing	1.120	0.571	15.667	0.004**	10.462	0.015*
Access to the internet during Physical Distancing	0.480	0.786	45.059	0.000**	44.569	0.000**
Ability to pay internet bills during Physical Distancing	1.117	0.572	86.062	0.000**	106.940	0.000**
Increasing internet bill during Physical Distancing	5.424	0.066	1.045	0.903	9.771	0.021*
Getting internet bill subsidy during Physical Distancing	2.288	0.319	11.208	0.024*	7.003	0.072

Social Safety Nets						
Access to cash or non-cash loans or assistance	0.294	0.683	9.958	0.041*	6.403	0.094
Assistance or loan help meet the needs of our family	4.602	1.000	7.567	0.109	4.346	0.226
Having a side job during Physical Distancing	0.501	0.779	17.115	0.002**	10.061	0.018*
Additional income from side job able to meet the needs of our family	1.833	0.400	12.849	0.012*	9.098	0.028*

Assets						

Status of residence	3.993	0.136	14.916	0.005**	20.856	0.000**
Savings	5.498	0.064	84.533	0.000**	205.177	0.000**
Valuable assets (gold and silver)	2.551	0.279	53.159	0.000**	45.532	0.000**
Immovable assets (land and buildings)	6.553	0.038*	16.259	0.003**	44.653	0.000**
Vehicle for daily activities (motorbikes or cars)	15.312	0.000**	12.348	0.015*	12.526	0.006**

Adaptive Capacity						
I have more than one source of income	6.393	0.041*	29.885	0.000**	32.775	0.000**
I have other skills that will be useful to get economic benefits during this Physical Distancing	6.687	0.035*	16.470	0.002**	0.930	0.818

Stability						

Numbers family members worked before Physical Distancing	1.444	0.237	2.332	0.054	1.701	0.165
Number family members lost their jobs/income during the Physical Distancing	1.753	0.174	15.981	0.000**	11.085	0.000**
Income condition during Physical Distancing	1.042	0.594	133.333	0.000**	53.791	0.000**
Spending conditions during Physical Distancing	8.338	0.015*	20.420	0.000**	0.635	0.888
Having health insurance	25.675	0.000**	92.300	0.000**	65.661	0.000**
Having insurance for assets (Motorbike, Car, House and others)	7.092	0.029*	43.222	0.000**	15.568	0.001**
Having debts	6.133	0.047*	18.843	0.001**	10.418	0.015
One of your family members have a credit card	1.200	0.549	31.741	0.000**	26.016	0.000**

** Significant at the 0.01 level; * Significant at the 0.05 level.

Table 4 presents the data of the spearman rank correlation between each item of financial aspects of economic security with a sample grouped in physical distancing patterns, sources of income, and estimated duration of economic resilience [1,2]. As similar to Tables 2 and 3 also indicate the significance of the relation between variable using the number of p-values <0.5 so that the data which presented in Table 3 is the number of p-values. The data indicate that there were almost no items significantly related to physical distancing patterns except for health insurance and insurance on movable property items. The data also indicate that there is a significant relationship between all items and family income sources except access to assistance/loans, having side jobs during physical distancing, the status of residence, and other skills that are economically useful. The same thing also happened in the estimated duration of economic resilience. Almost all items have a significant relationship with the estimated duration of economic resilience except for other skills items that are economically useful, spending patterns, and debt ownership. These findings indicate interesting characteristics of the people of Medan in dealing with physical distancing in the face of a COVID-19 global pandemic.

**Table 4:** Correlations Analysis of Economic Indicator to Pattern of Physical Distancing, Source of the Main Income and Economic Resilience.

	Pattern of Social/Physical Distance	Source of the family's main income	If Physical Distancing will continue to apply, how long will you and your family survive economically

Income	0.173	0.000**	0.000**
Income per-capita	0.066	0.000**	0.000**
Access to cash or non-cash loans or assistance	0.611	0.896	0.026*
Having a side job during Physical Distancing	0.481	0.566	0.001**
Status of residence	0.060	0.645	0.000**
Savings	0.170	0.000**	0.000**
Valuable assets (gold and silver)	0.203	0.000**	0.000**
Immovable assets (land and buildings)	0.613	0.003**	0.000**
Vehicle for daily activities (motorbikes or cars)	0.997	0.009**	0.002**
I have more than one source of income	0.249	0.000**	0.000**
I have other skills that will be useful to get economic benefits during this Physical Distancing	0.391	0.794	0.358
Income condition during Physical Distancing	0.339	0.000**	0.000**
Spending conditions during Physical Distancing	0.145	0.001**	0.707
Having health insurance	0.000**	0.000**	0.000**
Having insurance for assets (Motorbike, Car, House and others)	0.016*	0.001**	0.001**
Having debts	0.105	0.004**	0.092
One of your family members have a credit card	0.288	0.000**	0.000**

** Correlation is significant at the 0.01 level (2-tailed).

* Correlation is significant at the 0.05 level (2-tailed).

## 2 Experimental design, materials and methods

The data is collected from the household of Medan. Medan is the capital city of North Sumatra. The researchers choose Medan because of Medan is the 3rd largest city in Indonesia and have contributed to a 3% COVID-19 case in Indonesia. Despite only give a 3% contribution, Medan still has moderate risk in facing COVID-19 because its geographical position was accessible both nationally and internationally. Therefore, The Mayor of Medan and Governor of North Sumatra decide Medan to do Physical Distancing and closes the school, mosque, public area, and tourism area following the President of Indonesia Instruction. In turn, the decision brought the economic risk among household in Medan. Beside, Medan was appropriate to be an indicator of regulation in facing it a financial threat for the region outside Java island. Java is the island of the capital city which the spreadable of COVID-19 higher than non-java while in facing the physical distancing every region facing similar economic risk. So that, the impact of COVID-19 global pandemic on economic regulation should be different between java cities/districts and non-java cities/districts.

This data proposed the household perception regarding their economic resilience in facing the government instructions to carry out Physical Distancing to anticipate COVID-19 Global Pandemic. Data were collected two weeks after the instructions were first announced using a convenience sampling technique. This is intended to get an actual response after the respondent feels the impact of the application of physical distancing regarding their economic resilience. The unit of analysis is the household. So, the data describe economic resilience at the household level. The dataset was collected by a survey method with an electronic questionnaire. The use of electronic questionnaires is intended to reach a broad range of respondents during the physical distancing caused by the global COVID-19 pandemic. The uses of an electronic questionnaire may risk to the bias of responses. However, the behavioral research method has stated the possibility of using electronic questioner in order to face several conditions related to time, space, access, and financial limitation [3]. Besides, nowadays, communication technology, like smartphones, was a common device in which almost everybody in the city owns it [4,5]. To control the bias, we designed the questionnaire which needs household information, as explained before. Furthermore, to prevent the small range of economics classes of respondents who participated, we control it using the family income.

The instrument used in data collection was adapted from Alinovi, Mane, and Romano (2008) [6]. The instrument was translated and adapted so that understandable for the characteristics of respondents in Medan. The questionnaire also designed anonymously to ensure the privacy of respondents [3,7]. In addition, respondents were asked to fill out the questionnaire voluntarily to maintain the independence of respondents in giving their responses. Data that is captured includes several categories either nominal, ordinal, or interval refers to the appropriateness of indicators with the information that allows being captured. Determination of the type of data also refers to the recommendation of previous research. The electronic questionnaire was developed using Google Form and distributed massively to all social media networks. Questionnaire distribution was conducted from 12 to 20 April 2020 and 1096 responses were collected. The entire data is then screened and filtered so that it only leaves 867 data with respondents who are domiciled in Medan City. In order to make the data more informative, researchers conducted a descriptive statistical analysis, ANOVA, Kruskal Wallis, and the Spearman's Rank correlation. Analysis of the data provides valuable information related to the interrelation of each item and the pattern of economic resilience that the urban city household has as a consequence of the COVID-19 global pandemic. Researchers can then further test the data using more advanced analytical tools to produce more valuable information in the development of science and in developing strategic policies related to anticipating the economic vulnerability of the household due to the global pandemic disaster (Fig. 1a, Fig. 1a, Fig. 1a, Fig. 1a).

**Fig. 1a:**
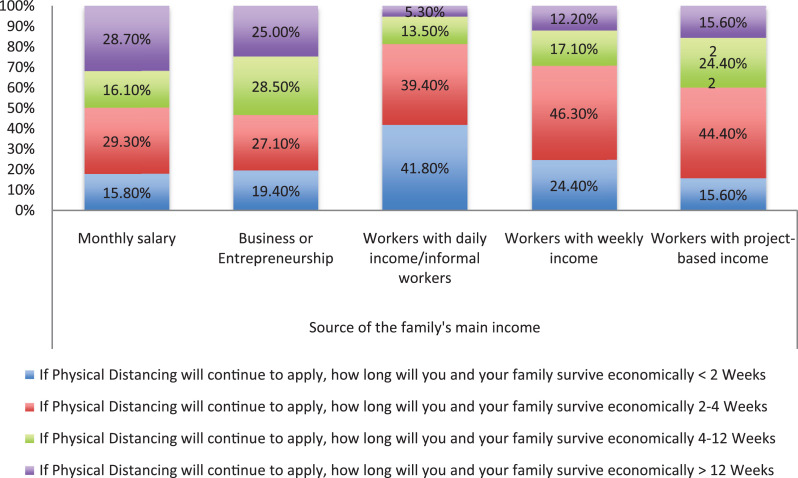
Cross-Tabulation between Economic Resilience and Source of the Family's Income.

**Fig. 1b:**
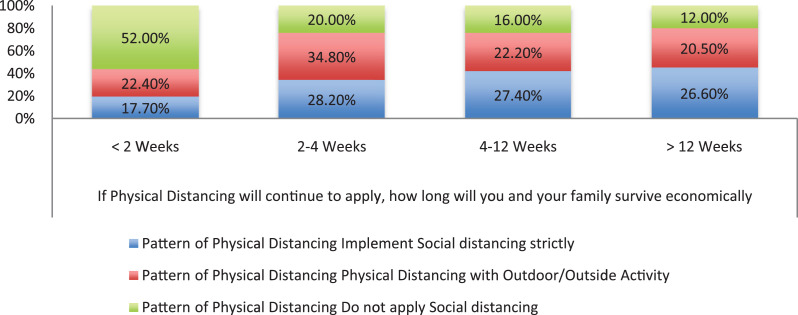
Cross-Tabulation between Economic Resilience and Pattern of Physical Distancing.

**Fig. 1c:**
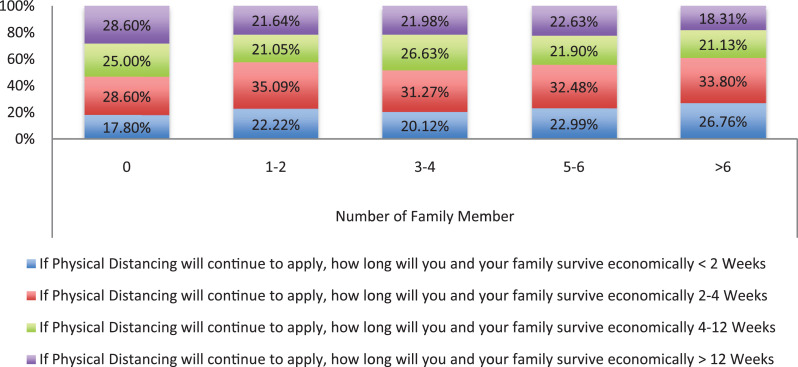
Cross-Tabulation between Economic Resilience and Number of Family Income.

**Fig. 1d:**
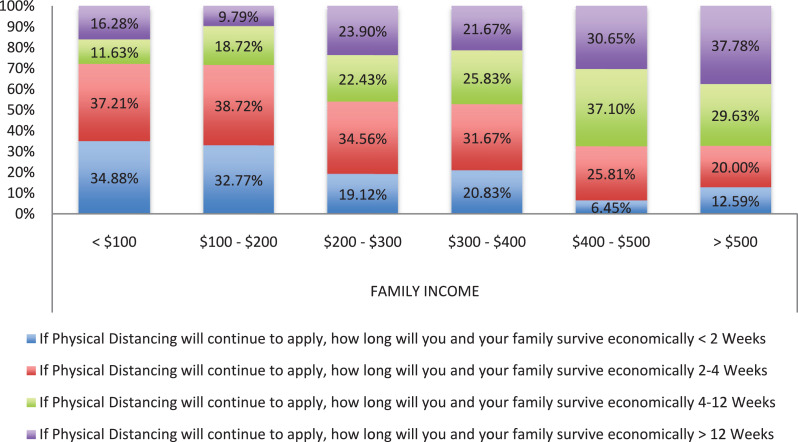
Cross-Tabulation between Economic Resilience and Family Income.

## Declaration of Competing Interest

None.

## Appendix Supplementary materials

Image, application 1
